# Image Fusion for Super‐Resolution Mass Spectrometry Imaging of Plant Tissue

**DOI:** 10.1002/advs.202512662

**Published:** 2025-11-19

**Authors:** Yuchen Zou, Shipeng Sun, Weiwei Tang, Bin Li

**Affiliations:** ^1^ State Key Laboratory of Natural Medicines and School of Traditional Chinese Pharmacy China Pharmaceutical University Nanjing 210009 China

**Keywords:** deep learning, image fusion, image super‐resolution, mass spectrometry imaging, plant tissue

## Abstract

Mass spectrometry imaging (MSI) is a vital tool in botanical research. Image fusion is introduced for resolution enhancement of MSI data from animal samples, but its application to plant MSI data resulted in unsatisfactory visualizations due to the distinct morphological characteristics of plant tissues. Herein, this study presents loss controlled residual network (LCRN), a workflow dedicated to the super‐resolution fusion of plant MSI data. The pipeline used a residual connection‐based neural network implemented with a novel loss metric called edge perceptual loss. Edge perceptual loss is developed for evaluating complex morphological information that can not be properly reflected by common image metrics, and its implementation in loss propagation is vital to the quality of the fusion result. Compared to existing deep learning‐based methods, LCRN is able to generate a high‐quality super‐resolution fusion image of extra high magnification (up to 20‐fold) that combined chemical and morphological information obtained from MSI and microscopy, respectively.

## Graphical abstract

A loss controlled residual network (LCRN) workflow is developed for super‐resolution fusion of plant mass spectrometry imaging data. LCRN uses a novel edge perceptual loss metric to preserve complex plant tissue morphology. LCRN achieves up to 20‐fold magnification while effectively combining chemical information from mass spectrometry with morphological details from microscopic images.

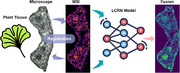

## 1 Introduction

Mass spectrometry imaging (MSI) has become one of the unique tools in the current analytical sandbox for its capabilities of spatially profiling hundreds to thousands of ions simultaneously through the whole sample, ranging from small metabolites to peptides and proteins. In botanical studies, MSI is utilized to snapshot the spatial distribution, accumulation, and transportation of key metabolic precursors, intermediates, and final products, facilitating the elucidation of plant functional genomes involved in complex secondary metabolism pathways.^[^ ^1^ ^]^ It has an irreplaceable place in today's analytical toolkit of botanical scientists.^[^ ^2^, ^3^ ^]^

Spatial resolution, detection sensitivity and specificity, and analysis speed are four fundamental aspects in MSI, determining the quality and accuracy of the obtained information in an MSI analysis.^[^ ^4^ ^]^ Among them, high spatial resolution is essential for investigating biological characteristics at the cellular level.^[^ ^5^ ^]^ However, higher spatial resolution results in a smaller pixel size, translating into fewer detectable analytes per scan, and thus requires higher sensitivity of the mass spectrometer and higher ionization efficiency.^[^ ^6^ ^]^ While biological MSI of sub‐10 micrometers is viable, the efforts and costs it requires are exponentially increased, with much higher requirements for the ionizability of the analytes. To date, these advanced MSI methods have focused more on the analysis of selected high‐abundance phospholipids possessing high ionizability in animal tissue sections,^[^ ^7^, ^8^, ^9^ ^]^ whereas high‐resolution MSI of many other metabolites is still in urgent need. On the other hand, MSI for plant samples has always been considered more difficult compared to animal tissue samples due to the complex and diverse structural composition of plant tissues.

To address the issue, image fusion has emerged as a viable option. This methodology originated from the disciplines of computer vision for the reconstruction of compressed images, which was soon introduced into medical imageology.^[^ ^10^ ^]^ Fusion workflow for MSI was first introduced in 2015, where partial least square (PLS) was used to generate sharpened images of selected ions from MSI data of rat brains and kidneys.^[^ ^11^ ^]^ With the emergence of multi‐omics and multi‐modalities analysis, image fusion as a data integration paradigm is gaining more and more attention.^[^ ^12^, ^13^, ^14^ ^]^ In addition to MSI image super resolution and out‐of‐sample prediction, image fusion now represents an attractive analytical paradigm that provides solid integrations of different modalities, such as morphological information from microscopy, spatial metabolomics from MSI, and spatial transcriptomics, granting scientists valuable insights from multiple perspectives.^[^ ^15^ ^]^ In terms of MSI, multiple deep learning‐based image fusion pipelines have been reported in the past 5 years. For example, a deep learning‐based fusion model was reported to enhance the spatial localization of small pharmaceutical compounds in rat brains.^[^ ^16^ ^]^ DeepFERE took it further by integrating the registration function into the fusion pipeline for the resolution‐enhanced MSI of rat brains.^[^ ^17^ ^]^ Notably, the utilization of a transfer learning strategy was reported recently, where models trained from optical super resolution were used in multiple MSI fusion tasks, ranging from rat brains to human colons.^[^ ^18^ ^]^

However, few implementations of these fusion methods have been reported in plant MSI. The effectiveness of these methods in plants is questionable due to the unpredictability brought by the drastic dissimilarity between animal and plant tissues. Herein, we proposed a fusion workflow based on deep learning dedicated to the fusion of MALDI MSI data and microscopic images of plant tissues, termed loss‐controlled residual network (LCRN), for combining data from two analytical modalities into a new form of information, suitable for complex morphology and high magnification factors (≈20 times magnification). Pipelines from existing works are being presented and compared to demonstrate the greater effectiveness of the proposed method. Notably, unlike typical samples from animal tissues, image fusion for plant MSI data has some unique issues that require specific optimizations, which have not been reported before. This article aims to explain and discuss these issues, in addition to providing a viable pipeline for the task of super‐resolution fusion of plant MSI and microscopic image data.

## 2 Results

### 2.1 Overview of LCRN

**Figure 1:**
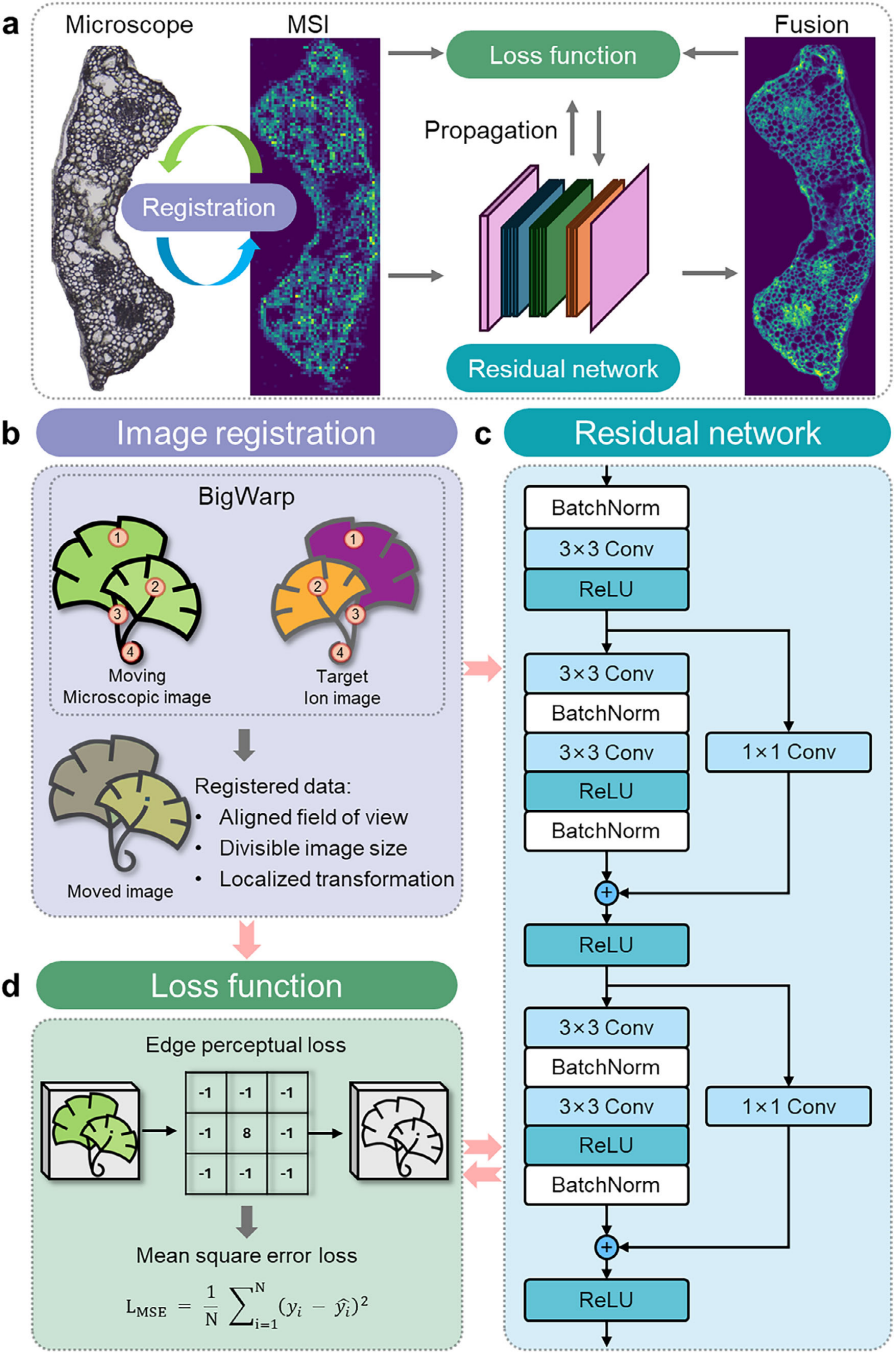
a) Overview of LCRN. b) BigWarp was used for image registration, which works by manually assigning pairs of corresponding dots in both images. The plugin would then apply localized transformation based on assigned dots and export the moving image with a specific spatial resolution and field of view of the target image. c) The residual network used in LCRN consists of two residual blocks and one initialization block. d) LCRN applied a special design loss function, which included MSE loss, and a novel loss metric named edge perceptual loss. The latter aimed to encompass morphological information in the training process, in addition to chemical information from MSE loss.

LCRN was designed from the very beginning. Unlike computer vision areas, the application scenario in MSI studies is on the contrary. MSI data is generally hard to access and low in numbers. Building a generic model with a huge amount of ground truth high‐resolution and high‐quality MSI is not practical, particularly for plant MSI data. Transfer learning via optical images is also not feasible due to the diverse staining methods used in different plant tissues. Therefore, a non‐generalization approach was adopted (**Figure** **1**a), where the model is trained to fit the correlation of the optical image array and a slice of MSI array (ion image). In LCRN, data from the microscope and MSI were first registered using BigWarp,^[^ ^19^ ^]^ a tool for manual landmark‐based deformable image alignment built as an ImageJ plugin (Figure 1b). Usually, a higher number and better precision of landmark pairs are preferred to minimize registration error and thereby improve fusion results. The registered data was then input into a residual‐connected network with two residual blocks. Each block consisted of two convolutional layers and a skip connection by a 1×1 convolutional kernel connecting the block input to the output (Figure 1c). For loss control, a custom loss function based on a novel developed loss metric was built (Figure 1d). Inspired by the concept of perceptual loss, this novel metric, termed edge perceptual loss, was calculated by using a convolutional layer with a designated kernel to extract the marginal features of the optical image and model output (Figure 1d). Then, the mean square error (MSE) of their marginal features is used to preserve the morphological information during the model training. For image registration, despite previous publications reporting several dedicated pipelines for such tasks, BigWarp was proven to be the most feasible choice for the current workflow. This tool is suitable for subtle, heterogeneous, and nonlinear deformation misalignment between data from the microscope and MSI, which was handled by manually assigning more than four pairs of dots (Figure S1, Supporting Information).

### 2.2 Visual Effectiveness of LCRN

**Figure 2:**
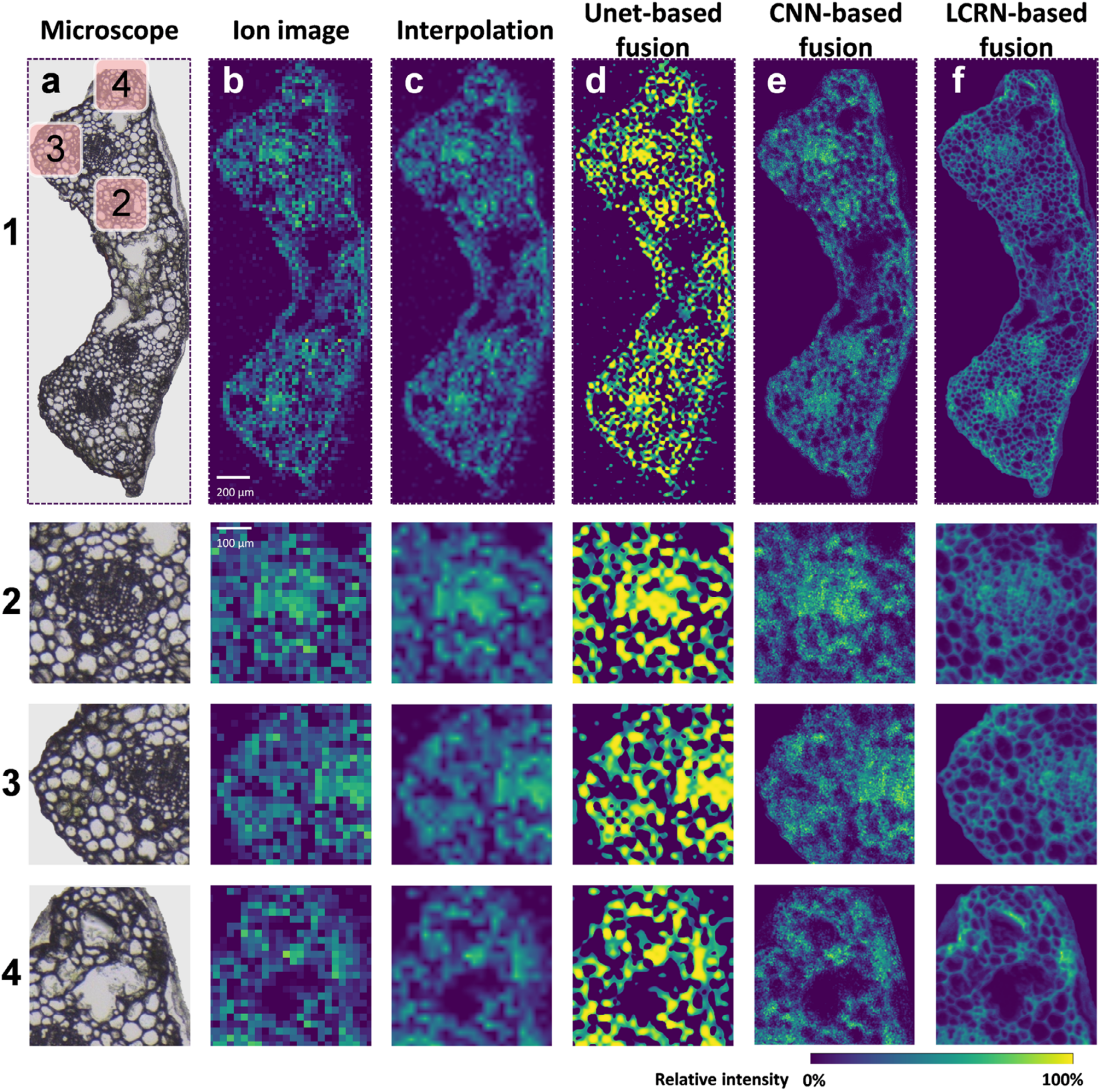
a) Microscopic image of the ginkgo leaf cross‐section. The image was registered using BigWarp. Region 2, 3, and 4 highlighted the vascular bundle, epidermis, and mesophyll, secretory cavity, respectively. b) Ion image of apigenin (m/z 269.044) in the ginkgo leaf cross‐section. Spatial resolution was equivalent to 20 µm. c) Bilinear interpolated image of b). d–f) Fusion results based on (a) and (b) using Unet‐based fusion method (d), CNN‐based fusion method (e), and LCRN (f), respectively. The method in (d) utilized Unet and multi‐stage training processes involving MSE loss, correlation loss, and reconstruction loss. The method in (e) utilized a three‐layer CNN with MSE loss. Both methods were adopted from previous reports. Spatial resolutions for (c–f) were equivalent to 1 µm.

Having established the fusion pipeline, LCRN was applied to enhance the visualization of the MSI of the ginkgo leaf cross‐section. *Ginkgo biloba* is an ancient plant, and its leaves are known for producing diverse secondary metabolites with significant pharmacological activities.^[^ ^20^ ^]^ The dataset was captured with a spatial resolution of 20 µm. A magnification factor of 20 is demonstrated, equivalent to 1 µm spatial resolution, which reaches the most advanced level of spatial resolution currently available in MSI. After data preprocessing,^[^ ^21^ ^]^ the dataset had a size of 135×49 pixels, and the 20× scaled optical image was 2700×980 pixels. The cross‐section of the ginkgo leaf revealed multiple types of cells: epidermis, vascular bundle, mesophyll, and secretory cavity (**Figure** **2**a). The vascular bundle consisted of many diverse‐sized cells (ROI 2 in Figure 2a), the epidermis was a normally sized one‐cell‐thick layer that surrounded the whole tissue (ROI 3 in Figure 2a), and the secretory cavity was the regularly shaped cavity located in both sides of the leaf section (ROI 4 in Figure 2a). The diversity of these variant structures posed a huge challenge for the fusion workflow. Figure 2b demonstrated the spatial distribution of apigenin (*m/z* 269.044), an active flavonoid distributed across the leaf section.^[^ ^22^ ^]^ These tissues were visible under the microscope but not in the ion image (Figure 2b), even after interpolation (Figure 2c). All applications of fusions resulted in improvements in image sharpness but led to different visualizations. Unet method enhanced the sharpness of the interpolated image but lost all morphological details (Figure 2d). Convolutional neural network (CNN) preserved relatively more morphological information but produced serious artifacts that disoriented most cell segments (Figure 2e). It was only in the LCRN fused image where the hotspots representing concentrations of the ions as well as the optical features, were assigned to specific regions and cells with much clarity, simplifying the process of inspection (Figure 2f; Figure S2, Supporting Information). LCRN revealed clear morphological details of the leaf section from the microscopic image compared to other methods (Figure 2c–f; Figure S2, Supporting Information), including vascular bundles and cell segments of the epidermis and mesophylls, and reflected the localized hotspot according to ion distribution as well as the color intensity of the optical image.

**Figure 3:**
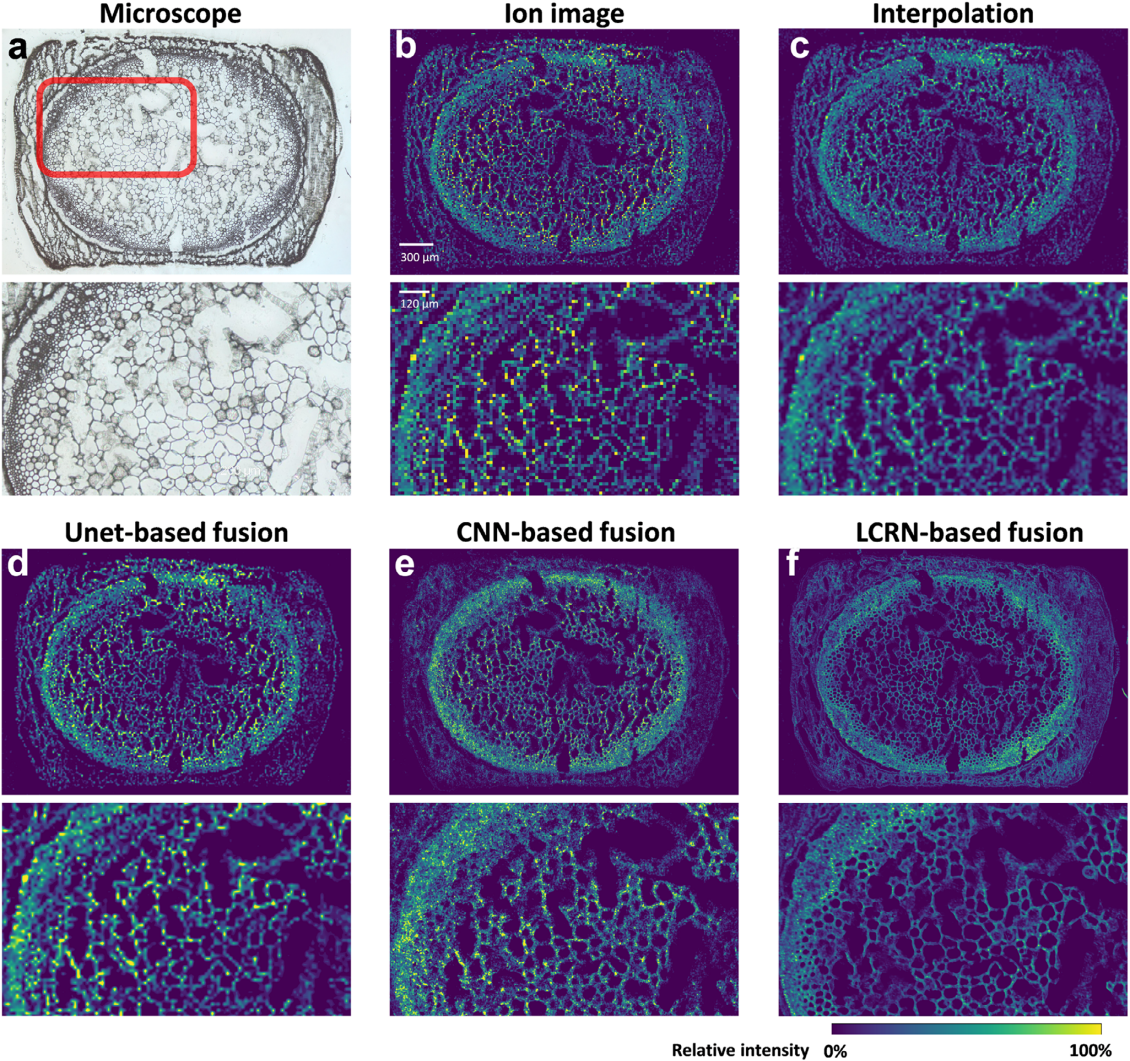
a) Microscopic image of the mint stem cross‐section. The image was registered using BigWarp. The red box highlighted a representative area of the diverse morphologies with enlarged views below. b) Ion image of *m/z* 669.165 in the mint stem cross‐section. Spatial resolution was equivalent to 12 µm. c) Bilinear interpolated image of (b). (d–f) Fusion results based on (a) and (b) using Unet‐based fusion method (d), CNN‐based fusion method (e), and LCRN (f), respectively. The method in (d) utilized Unet and a single‐stage training process with MSE loss. The method in (e) utilized a three‐layer CNN with MSE loss. Spatial resolutions for (c–f) were equivalent to 1.2 µm.

We also applied our method to another dataset of stem cross‐sections of mint (**Figure** **3**; Figure S3, Supporting Information) to demonstrate the universality of LCRN. The dataset was obtained with a spatial resolution of 12 µm, and a magnification factor of 10 is demonstrated, equivalent to 1.2 µm spatial resolution and an image size of 1800×2550 pixels. Due to the ≈1.66 times increase in spatial resolution of ion image of mint stem compared to the ion image of Ginkgo leaf, the ion image has similar morphological features to those in the microscopic image, but the ion image was not able to reveal the small cells near the cambium and phloem area due to the limitations of spatial resolution (Figure 3a,b). The visualization of Unet and CNN methods was similar to that of Ginkgo. Unet provided results similar to interpolation (Figure 3c) but with much more sharpening (Figure 3d), and CNN brought artifacts and lost cell segments (Figure 3e). On the contrary, LCRN was able to surpass the above methods and reveal this area with much clearer cell segments (Figure 3f).

### 2.3 Selection of the Evaluation Metric

While designing the proposed fusion workflow, there were two core choices for the structure of the method: the network architecture and the loss function. Despite previous methods reported to be viable in animal samples, those methods did not produce satisfactory fusion results for plant MSI (Figures 2d,e and 3d,e), indicating their designs were not suitable for the fusion task of plant MSI. Based on extensive tests, residual network (ResNet), which was developed based on CNN, was finally selected as the basic architecture for our proposed method. It adds skip connections that pass a direct mapping between inputs and outputs in each block, improving performance drastically with little addition to the calculation burden. ResNet has been one of the most influential architectures of neural networks since its emergence, dominating the benchmark for image classification on ImageNet. Our benchmarking tests reveal their superior fitting capabilities, compared to other network structures used in previous fusion reports (Figure S4, Supporting Information).

However, fitting capabilities do not directly translate into better results in the current task. The neural network needs a quantitative metric to work properly, which reflects the quality of the fused image. A typical evaluation metric implemented as a loss function in previous fusion pipelines is the MSE between the interpolated ion image and the model output. This metric is representative of the overall intensity distribution but tends to neglect the texture change that has a huge visual impact. More importantly, due to the lack of ground truth data as the target, the deep learning network would fit the input image into the interpolated ion image, ultimately given enough training time. This might work in low magnification fusion of animal samples because the low magnification interpolation did not bring too many errors in these cases. However, in the current task, high‐magnification interpolation brought excessive disparity. Therefore, a second metric calculated from the optical image was necessary so that it could represent the human visual sense of the image as comprehensively as possible.

**Figure 4:**
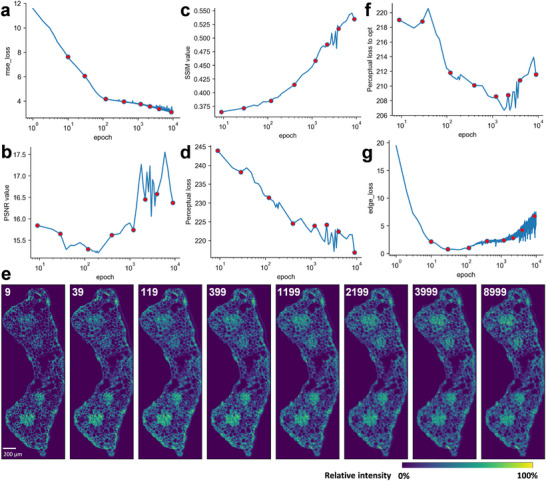
a) MSE loss value per training epoch. b–d) PSNR value (b), SSIM value (c), and perceptual loss value (d) of each checkpoint image. e) Part of the checkpoint images during the training process. The whole set of checkpoint images can be found in Figure S5 (Supporting Information). The sequence of the presented checkpoints was correspondent with the red dots in all other plots. f) Perceptual loss value to the input of the microscopic image. g) Edge perceptual loss value per training epoch. For all the plots, please note that the epoch axis is logarithmic in order to linearly approximate the drop of MSE loss. The trends of each metric did not change due to the use of a logarithmic axis (Figure S6, Supporting Information). Plots in (b–d) and (f) were based on the whole set of checkpoint images (Figure S5, Supporting Information). Plots in (a) and (g) were based on the complete training process.

Inspired by the concept of perceptual loss,^[^ ^23^ ^]^ edge perceptual loss was invented, which could fully reflect the human visual sense trends of the image. Several common metrics were compared to the newly developed edge perceptual loss (**Figure** **4**). Peak signal‐to‐noise ratio (PSNR) is similar to MSE but is also impacted by the maximum intensity of the image. Another common metric is the structural similarity index (SSIM), which focuses on the structure, brightness, and contrast of the image to simulate the sense from human visuals. However, the calculation of SSIM is indiscriminate throughout the image, possibly making its evaluation distinct from actual human eyes, which have different sensitivity regarding flat areas and fine‐texture areas. Perceptual loss was also tested. This metric uses certain layers of established CNN (for example, AlexNet and VGG) to extract abstract features from the image and calculate their differences. These metrics were tested in a training session using a residual network and MSE loss. Checkpoints were saved and evaluated with different metrics (Figure S5, Supporting Information). Overall, all metrics had the same alternating trend toward the ideal direction as the MSE value with fluctuations (Figure 4a–d). However, the checkpoint images demonstrated differently (Figure 4e). The very first checkpoint (9) has not fully captured the information from the ion image, and the checkpoint (39) seemed to reach a balance. After this one, the images gradually gained fuzzy patterns as training proceeded, which ultimately disoriented the whole image and lost morphological details (Figure 4e). Although the actual mechanism of deep learning is a black box, this disparity could be attributed to the loss of morphological information from the input image. Additionally, metrics calculated based on microscopic images were tested (Figure 4f; Figure S6, Supporting Information), which still show similar trends as Figure 4a–d. The plot of edge perceptual loss fitted the manual evaluation of the checkpoint images (Figure 4e,g), indicating this metric was suitable for representing human visual sense in the current task.

### 2.4 Back Propagation Method

**Figure 5:**
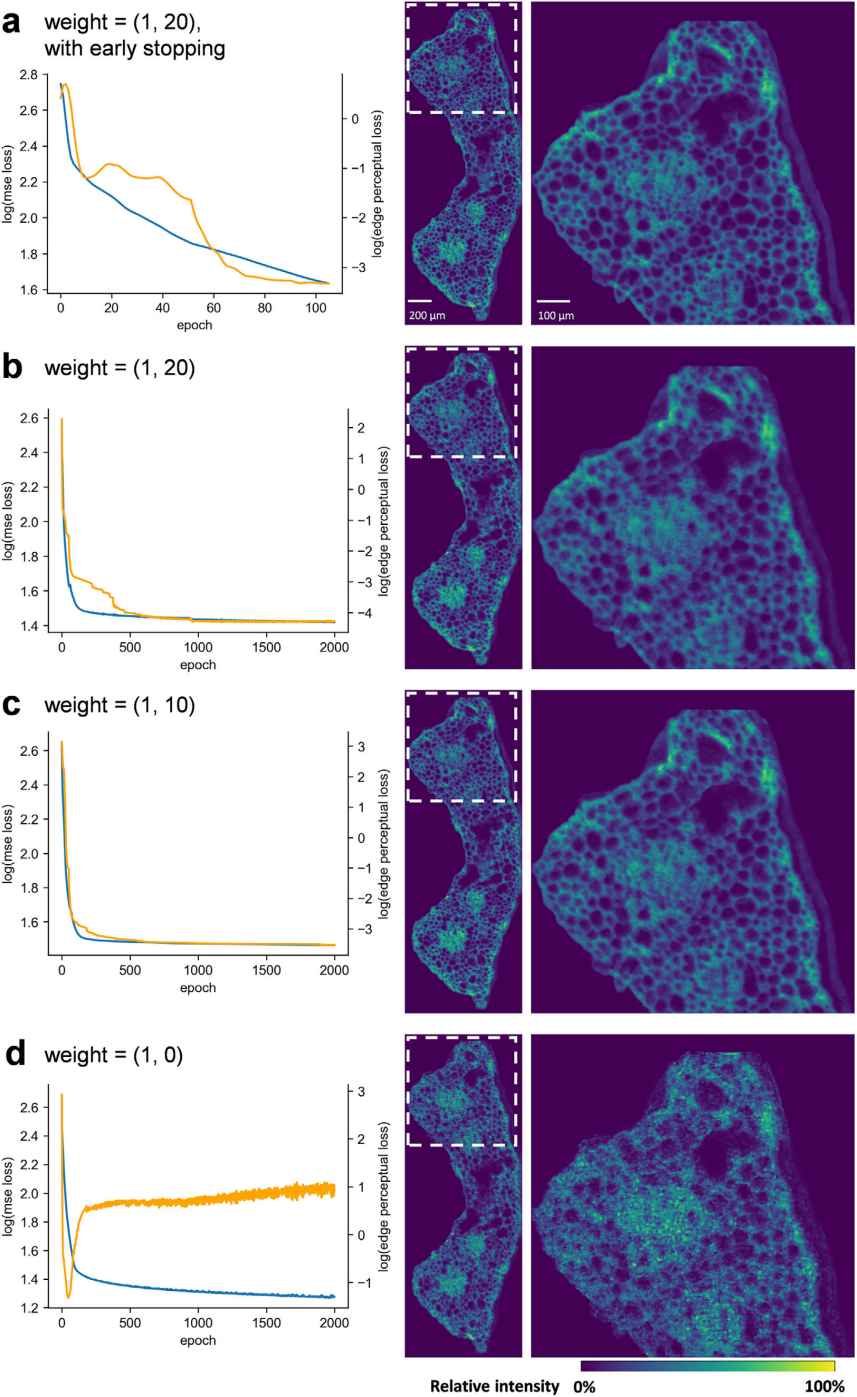
a–d) Output results from different training settings and changes of MSE loss (blue line) and edge perceptual loss (orange line) during training. Note that the value was logarithmic. Weight was represented as (multiplier of MSE loss, multiplier of edge perceptual loss). The (1, 0) weight in d) indicated that only MSE loss was propagated, while edge perceptual loss was calculated and recorded, but did not participate in loss propagation.

Although edge perceptual loss was found to be representative of human visual sense, it still needed to be implemented in the training process in addition to the existing MSE loss. A common strategy was simply adding two loss metrics, but the downside is self‐evident: the disparity of orders of magnitude between two losses would make one of the loss metrics nearly obsolete. Applying weighing on different loss metrics could be an obvious fix, but this method also has potential issues due to different convergence rates of the metrics. The design of this type of multi‐loss deep learning, also known as multi‐task learning, is still under active research in the cutting‐edge front of deep learning disciplines. Therefore, based on the altering trends of edge perceptual loss, a weighted loss additive sum plus an early stopping mechanism was adopted. Basically, if the edge perceptual loss was not optimized along with the overall loss for a certain number of epochs, the training would end. Due to different convergence rates of the two metrics, the overall loss continued to decrease as the training proceeded, despite the fusion image having started to obtain fuzzy patterns. In a training session of 2000 epochs, which was excessive, the implementation of early stopping was able to pull off the training before the appearance of fuzzy patterns (**Figure** **5**a), compared to additive sum methods with different weights (Figure 5b–d). Without early stopping, a proper weight might still be able to generate a satisfactory fusion result but would require additional procedures to seek an optimized number of training epoch, because prolonged training might result in increased fuzziness in the fusion result (Figure 5b). Figure 5c,d indicated the great impact of introducing edge perceptual loss into the loss propagation. With the same training epochs and identical network structure, the addition of edge perceptual loss greatly preserved the morphology while still being able to keep minimizing MSE loss. This result further confirmed that edge perceptual loss was highly suitable for the fusion workflow of plant MSI data.

## 3 Discussion

To date, there have been around a dozen publications reporting methods for the fusion of MSI and optical images. These methods are diverse in terms of the algorithms and implementations, but they both follow a similar underlying scheme, which is generating a new image (a data array) that has high similarities to both optical image (which is usually a single image) and MSI (which could be a slice, multiple slices, or embeddings). This could be achieved by machine learning, such as multivariate regression and manifold learning^[^ ^11^, ^13^ ^]^ or deep learning, such as CNN or generative adversarial network (GAN).^[^ ^16^, ^17^, ^18^ ^]^ As deep learning brought more flexible and adaptive calculation frameworks, data fusion was also considered as a key methodology to the realization of multi‐omics or multi‐modality analysis, which combines metabolomics, transcriptomics, spectroscopy, etc. In botanical research, this approach is able to predict the localization of specialized metabolites within specific cell types or tissues, enable accurate mapping of metabolites within well‐defined cellular structures, as well as link chemical information to the context of other analytical aspects, such as transcriptomics and morphologies. By overcoming the trade‐off between spatial resolution and molecular coverage, image fusion allows researchers to precisely correlate metabolite localization with physiological processes like biosynthesis and transport, offering a more comprehensive understanding of plant metabolism and function at the single‐cell or even subcellular level. This information is impossible to obtain from bulk tissue analysis or current MSI but is essential to deciphering metabolic pathways of substances of interest inside plants, as only at the single‐cell level can the metabolic diversity of cells from the same tissue but in different development stages be revealed.

However, few studies have implemented these fusion methods on plant MSI data, for reasons that could be attributed to the intrinsic differences between plant and animal tissues. For animal tissues, partitions of cells of different types are more likely to be grouped, making the segmentation relatively more obvious. Unlike animal tissues, despite plants usually having much larger cells than animals, the distribution patterns of certain types of cells could be highly diverse. Some are grouped, such as cambium cells, while some appear to be scattered or in a highly spread‐out pattern, such as parenchymal cells. The diverse physical properties (including light transmittance, moisture content, color, etc.) of the plant tissue section are another potential issue that complicates the task. Animal tissues are generally more consistent in light transmittance, as different regions with highly differential chemical profiles still have similar transparency under a microscope. Plant tissue sections under microscopes are visualized with high contrast, as their compositions are much more heterogeneous. These differences in image patterns may have a huge impact on the feasibility of the methods. Although a simpler network was previously reported to be viable in the fusion of mammalian tissue MSI data, our tests on plant samples, which were more complex and irregularly shaped, indicated the necessity of optimization while designing fusion pipelines. In this study, the use and implementation of edge perceptual loss was proved to make a huge impact on the model training and fusion. This newly developed metric could represent the human visual sense very well, but it still cannot detect the gradually concentrated fuzzy patterns as the training proceeds excessively, indicating further room for improvement.

In the presented example, we proposed two core optimizations specifically designed for plant MSI fusion. ResNet‐like structure was adopted for the first time in the MSI fusion task, providing more powerful fitting capabilities with less cost on calculation efficiency. A novel metric termed edge perceptual loss was also developed to preserve complex plant morphology in fusion results. Using LCRN, predictive high‐resolution images were generated, which had both spatial chemical information and high‐resolution morphological information. Data fusion is the representative methodology in the current trend of deep learning‐based multimodality analysis paradigm. However, it is still under development for MSI‐based spatial metabolomics, especially in botanical studies. This work aims to provide a simple and viable strategy for the fusion of plant MSI data and hopes to lay the foundation for a more sophisticated and standardized establishment of an MSI data fusion strategy.

## 4 Experimental Section

### Sample Preparation for MSI

For both types of samples (ginkgo leaf and mint stem), fresh tissues were immediately embedded in 10% gelatin (wt/vol) solutions after collection. Initially, tissues were kept in Tissue‐Tek cryomolds (25 × 20 × 5 mm), and the gelatin solution was poured over them to embed the tissues. Thereafter, the molds were transferred to a −80 °C freezer for 30 min to form a solid block. For cryo‐sectioning, the sample blocks were directly fixed on the sample holder of a cryostat (Leica, Germany), using deionized water as the adhesive. Sections of 16 µm thickness were obtained at −20 °C and thaw‐mounted on indium tin oxide‐coated glass slides for immediate imaging measurements. To avoid condensation, the tissue sections were dehydrated in a vacuum desiccator for ≈10 min prior to matrix application. A Zeiss Axio M2 microscope (Zeiss, Germany) was used to obtain optical images of the sections.

### Matrix Application for MSI

A laboratory‐constructed automated pneumatic‐assisted matrix application system was used for the uniform application of MALDI matrix solution. The matrix application system and coating procedure were similar to our previously published work with some modifications. For negative mode MALDI, 10 mg mL^−1^ 9‐AA dissolved in methanol:water (9:1, vol/vol) was applied. For homogenous deposition onto the leaf samples, the nebulizer was held 3 cm above the sample and oscillated over the plate 100 times. The flow rate was set to 6‐8 mL hr^−1^ and gas pressure to 50 psi, to deliver and nebulize the matrix solution, respectively.

### MALDI MSI Instrument

MALDI imaging measurements were performed on an atmospheric pressure (AP‐SMALDI AF (TransMIT GmbH) ion source coupled to an Orbitrap mass spectrometer (Q Exactive HF; Thermo Fisher Scientific, Waltham, MA, USA).

### Data Processing—MSI Data Processing

For ginkgo leaf sample, the data obtained were exported into imzML format with a spatial resolution of 135 × 49 (height × width) from the instrument. Further preprocessing was conducted with shinyCardinal v0.3.0, a web‐based distribution of the R package Cardinal, where ion images were inspected and exported into csv file for a Python coding environment. The same procedures were also applied to mint sample data, which had a spatial resolution of 215 × 255. Interpolation of the ion image was done using OpenCV in Python. The interpolation option was set to “INTER_LINEAR”, which used a bilinear interpolation algorithm.

### Data Processing—Image Registration

The optical images obtained from ginkgo leaf sample and mint stem sample were exported from the microscope workstation into a TIFF file with the spatial resolution of 11939 × 4837 and 2539 × 2644, respectively. The image was imported into Fiji, an ImageJ distribution, together with a representative ion image exported by shinyCardinal and enlarged to the magnified size (original size x magnification factor). BigWarp, a plugin of Fiji, was used to align two images and alter the size of the optical image. An exemplar operation is shown in Figure S1 (Supporting Information).

### Fusion Workflow

Compared to conventional CNN, residual block used here added a skip connection linking the input of the block to the output. Mean squared error loss was used, calculated as below:

(1) $$L_{\mathrm{MSE}}=\frac{1}{N}\sum_{i=1}^{N}{\left(y_{i}-\hat{y_{i}}\right)}^{2}$$

where *y_i_* and $\hat{y_{i}}$ are the i‐th pixel of the output and the interpolated target ion image, respectively.

For edge perceptual loss, a designated 3×3 convolutional kernel was used to extract edge features of the input and the target. The edge perceptual loss was then represented by the MSE loss of the extracted features:

(2) $$L_{\mathrm{edge}}=\frac{1}{M}\sum_{i=1}^{M}{\left(K⊛y_{i}-K⊛X_{i}\right)}^{2}$$

where *K* is the designated 3×3 convolutional kernel to extract edge features of the inputs, $X_{i}$ represents the input microscopic image, and ⊛ denotes the convolution operation. The total loss that backwards was the additive sum of the loss times its respective weight:

(3) $$L=w_{1}\times \;L_{\mathrm{MSE}}+w_{2}\times \;L_{\mathrm{edge}}$$

### Training

The training was done on an RTX 4090 setup with 24 GB video RAM. The initial number of epochs was set to 2000, which served as the upper limit for the early stopping. Adam optimizer was employed with default settings. Learning rate was set to 0.0003 for both datasets. An early stopping mechanism was implemented, where the default stopping criteria were 5 total counts of loss increase (can be altered as a hyperparameter).

The network used in the fusion of the gingko and mint dataset was a residual‐connected network with two residual blocks followed by an initial block. For each block, the number of channels was 3 (initial input), 64 (initial output and first residual block input), 32 (first residual block output and second residual block input), and 1 (second residual block output), respectively. With one RTX 4090, the iteration speed for the ginkgo leaf dataset and the mint dataset was 14.3 and 8.0 iterations per second, respectively.

## Conflict of Interest

The authors declare no conflict of interest.

## Author Contributions

Y.Z. performed conceptualization, investigation, methodology, validation, and wrote the original draft. S.S. performed MSI. W.T. and B.L. performed conceptualization, supervision, and wrote, reviewing and edited the final manuscript.

## Supporting information



Supporting Information

## Data Availability

The data that support the findings of this study are available in the supplementary material of this article. Codes are available at https://github.com/codexyster/LCRN‐pr.

## References

[1] Y. Zou , W. Tang , B. Li , Trends Plant Sci. 2025, 30, 69. 39341734 10.1016/j.tplants.2024.08.002

[2] Z. Yin , W. Huang , K. Li , A. R. Fernie , S. Yan , Plant J. 2024, 119, 2168. 38990529 10.1111/tpj.16924

[3] Z. Yin , W. Huang , A. R. Fernie , S. Yan , Trends Plant Sci. 2023, 28, 250. 36411181 10.1016/j.tplants.2022.10.009

[4] S. Schulz , M. Becker , M. R. Groseclose , S. Schadt , C. Hopf , Curr. Opin. Biotechnol. 2019, 55, 51. 30153614 10.1016/j.copbio.2018.08.003

[5] H. Zhang , D. G. Delafield , L. Li , Nat. Methods 2023, 20, 327. 36899158 10.1038/s41592-023-01774-6

[6] J. Soltwisch , H. Kettling , S. Vens‐Cappell , M. Wiegelmann , J. Müthing , K. Dreisewerd , Science 2015, 348, 211. 25745064 10.1126/science.aaa1051

[7] M. Kompauer , S. Heiles , B. Spengler , Nat. Methods 2017, 14, 90. 27842060 10.1038/nmeth.4071

[8] M. Niehaus , J. Soltwisch , M. E. Belov , K. Dreisewerd , Nat. Methods 2019, 16, 925. 31451764 10.1038/s41592-019-0536-2

[9] J. Soltwisch , B. Heijs , A. Koch , S. Vens‐Cappell , J. Höhndorf , K. Dreisewerd , Anal. Chem. 2020, 92, 8697. 32449347 10.1021/acs.analchem.0c01747

[10] A. P. James , B. V. Dasarathy , Inf. Fusion 2014, 19, 4.

[11] R. Van De Plas , J. Yang , J. Spraggins , R. M. Caprioli , Nat. Methods 2015, 12, 366. 25707028 10.1038/nmeth.3296 PMC4382398

[12] L. Bergenstråhle , B. He , J. Bergenstråhle , X. Abalo , R. Mirzazadeh , K. Thrane , A. L. Ji , A. Andersson , L. Larsson , N. Stakenborg , G. Boeckxstaens , P. Khavari , J. Zou , J. Lundeberg , J. Maaskola , Nat. Biotechnol. 2022, 40, 476. 34845373 10.1038/s41587-021-01075-3

[13] T. Smets , T. De Keyser , T. Tousseyn , E. Waelkens , B. De Moor , Anal. Chem. 2021, 93, 3452. 33555194 10.1021/acs.analchem.0c04759

[14] X. Tian , B. Xie , Z. Zou , Y. Jiao , L.‐E. Lin , C.‐L. Chen , C.‐C. Hsu , J. Peng , Z. Yang , Anal. Chem. 2019, 91, 12882. 31536324 10.1021/acs.analchem.9b02792 PMC6885010

[15] G. Wang , B. Heijs , S. Kostidis , R. G. J. Rietjens , M. Koning , L. Yuan , G. L. Tiemeier , A. Mahfouz , S. J. Dumas , M. Giera , J. Kers , S. M. Chuva de Sousa Lopes , C. W. van den Berg , B. M. van den Berg , T. J. Rabelink , Cell Stem Cell 2022, 29, 1580. 36332571 10.1016/j.stem.2022.10.008

[16] Z. Liang , Y. Guo , A. Sharma , C. R. McCurdy , B. M. Prentice , Anal. Chem. 2024, 96, 11869. 38982936 10.1021/acs.analchem.4c01553 PMC11649305

[17] L. Guo , J. Zhu , K. Wang , K.‐K. Cheng , J. Xu , L. Dong , X. Xu , C. Chen , M. Shah , Z. Peng , J. Wang , Z. Cai , J. Dong , Anal. Chem. 2023, 95, 9714. 37296503 10.1021/acs.analchem.3c02002

[18] T. Liao , Z. Ren , Z. Chai , M. Yuan , C. Miao , J. Li , Q. Chen , Z. Li , Z. Wang , L. Yi , S. Ge , W. Qian , L. Shen , Z. Wang , W. Xiong , H. Zhu , Nat. Mach. Intell. 2023, 5, 656.

[19] J. A. Bogovic , P. Hanslovsky , A. Wong , S. Saalfeld , in 2016 IEEE 13th International Symposium on Biomedical Imaging (ISBI) , Prague, Czech Republic, April 2016.

[20] B. Li , E. K. Neumann , J. Ge , W. Gao , H. Yang , P. Li , J. V. Sweedler , Plant Cell Environ 2018, 41, 2693. 29966033 10.1111/pce.13395

[21] Y. Dong , U. Heinig , Mass spectrometry imaging data analysis with ShinyCardinal, Research Square 2024, 10.21203/rs.3.rs-4072606/v1.

[22] B. Salehi , A. Venditti , M. Sharifi‐Rad , D. Kręgiel , J. Sharifi‐Rad , A. Durazzo , M. Lucarini , A. Santini , E. B. Souto , E. Novellino , H. Antolak , E. Azzini , W. N. Setzer , N. Martins , Int. J. Mol. Sci. 2019, 20, 1305. 30875872 10.3390/ijms20061305 PMC6472148

[23] R. Zhang , P. Isola , A. A. Efros , E. Shechtman , O. Wang , in Proceedings of the IEEE Conference on Computer Vision and Pattern Recognition (CVPR) 2018, pp. 586–595.

