# Supplementary material for: Image Fusion for Super‐Resolution Mass Spectrometry Imaging of Plant Tissue
Source: Adv Sci (Weinh). 2025 Nov 19;13(7):e12662. doi: 10.1002/advs.202512662 (PMC12866858; doi:10.1002/advs.202512662)
Supplement: Supplementary file 1 — Supporting Information [file ADVS-13-e12662-s001.docx]

Supporting information

Image fusion for super-resolution mass spectrometry imaging of plant tissue

Yuchen Zou, Shipeng Sun, Weiwei Tang*, Bin Li*

State Key Laboratory of Natural Medicines and School of Traditional Chinese Pharmacy, China Pharmaceutical University, Nanjing, 210009, China

* Corresponding authors:

E-mail: weiweitang@cpu.edu.cn (W. Tang); binli@cpu.edu.cn (B. Li)

# Supplementary figures and tables


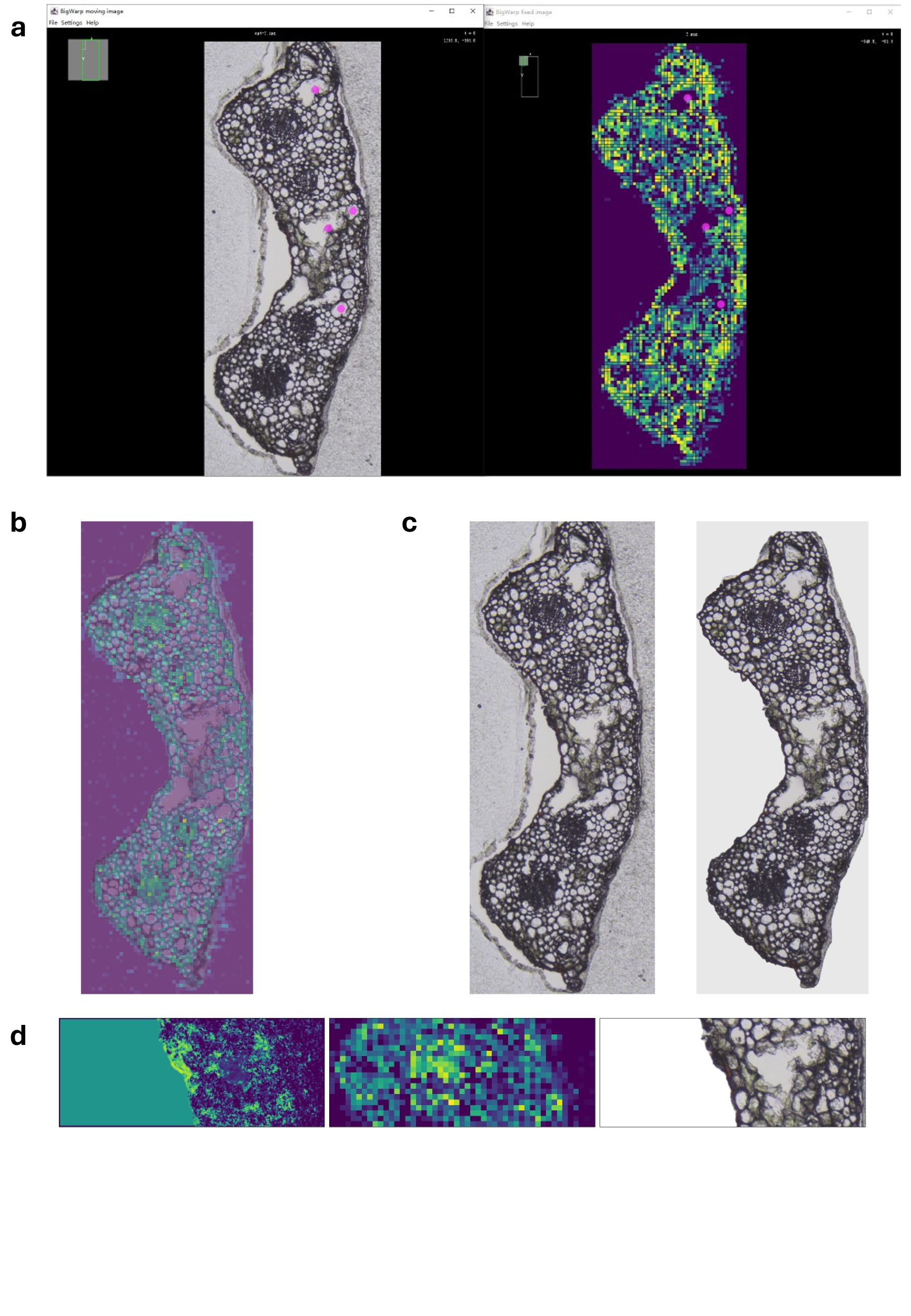


Figure S1. a) BigWarp user interface. The dots were assigned manually, and the plugin would transform the optical image to match the ion image according to the assigned dots. b) Overlapping of ion image and registered microscopic image. c) Microscopic image before (left) and after the registration (right). d) Fusion image (left) based on major misalignment of ion image patch (middle) and microscopic image patch (right).


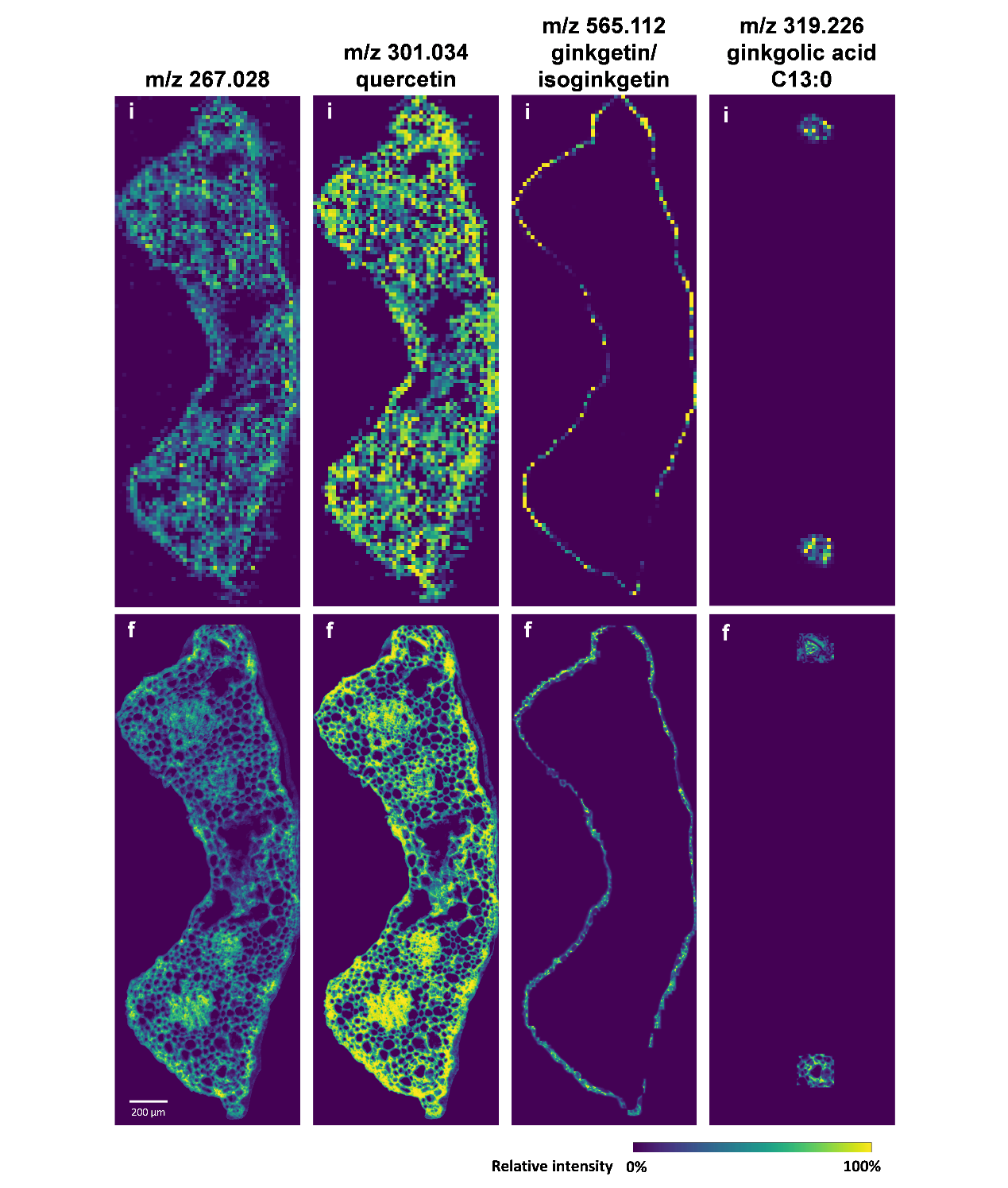


Figure S2. Ion images (label ‘i’) and LCRN fusion results (label ‘f’) of other ions from the ginkgo leaf dataset.


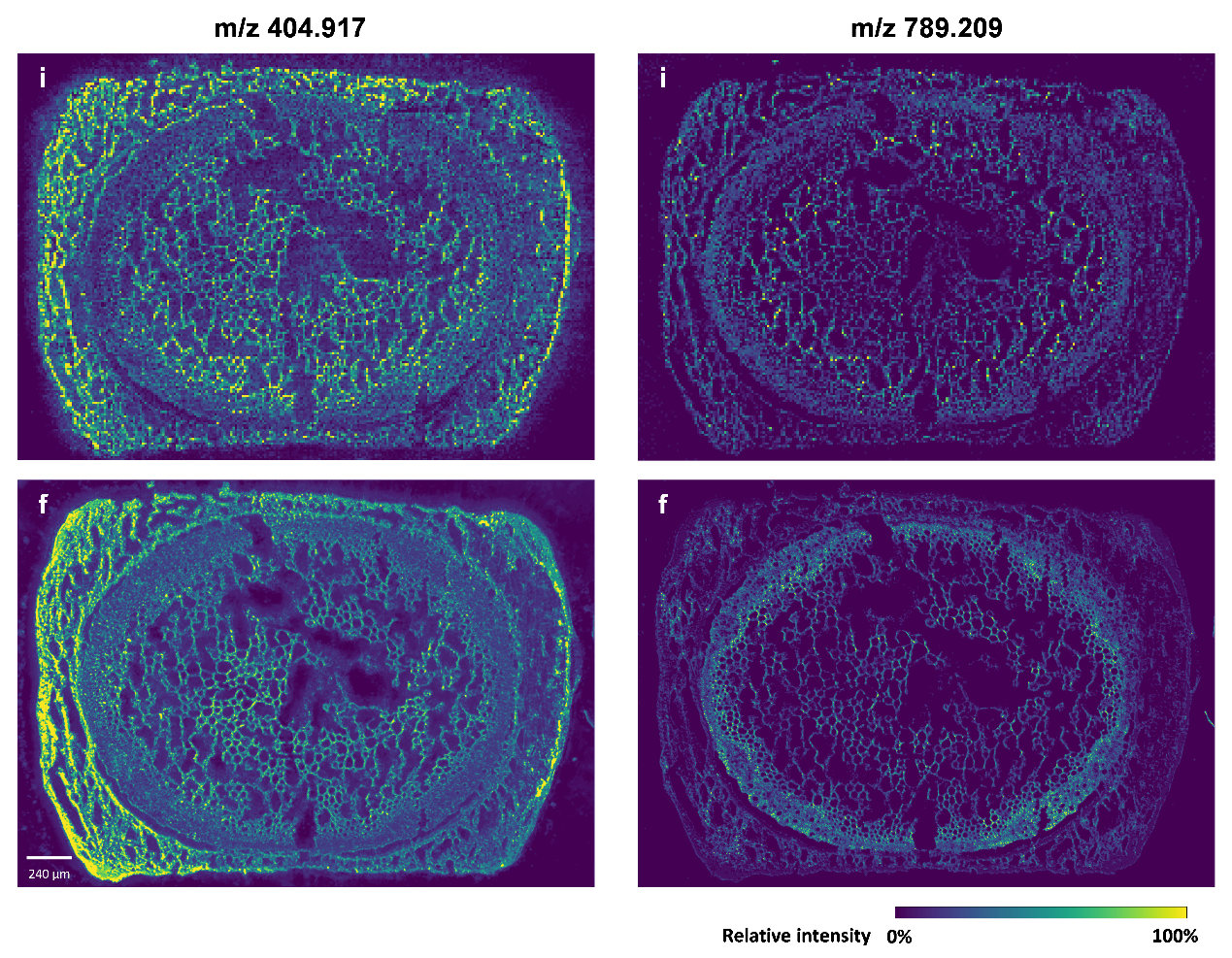


Figure S3. Ion images (label ‘i’) and LCRN fusion results (label ‘f’) of other ions from mint stem dataset.


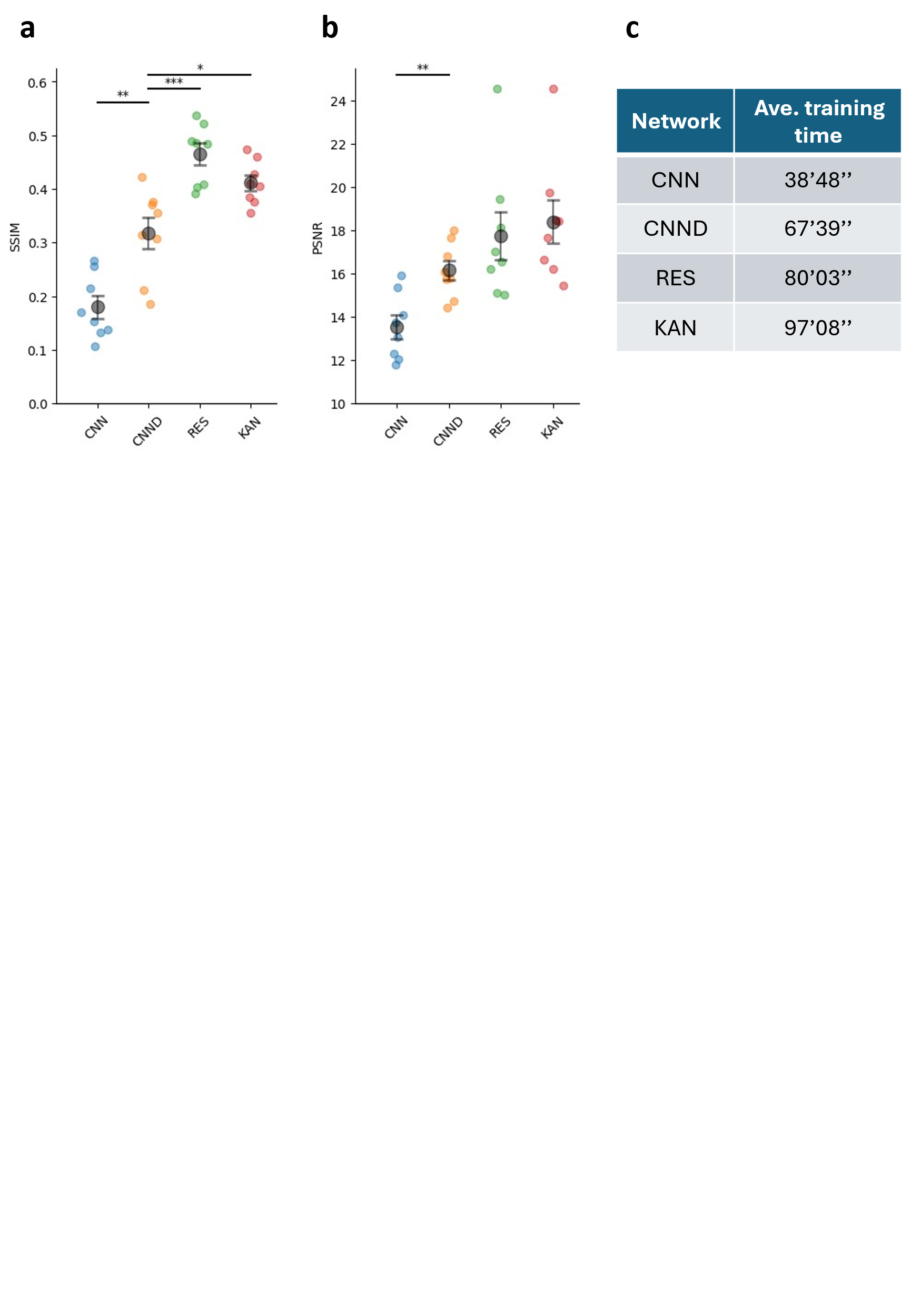


Figure S4. Fitting capability of networks with MSE loss represented by SSIM and PSNR. PSNR is proportional to the 10 logarithms of the reciprocal of MSE. For each network, ‘CNN’ is a three-layer convolutional neural network. ‘CNND’ is a five-layer neural network. ‘RES’ is a residual network consisting of two residual blocks. ‘KAN’ is a three-layer convolutional neural network, with the middle layer based on the Kolmogorov-Arnold network. The test was based on eight different ion images. ***: p<0.005; **: p<0.01; *: p<0.05.


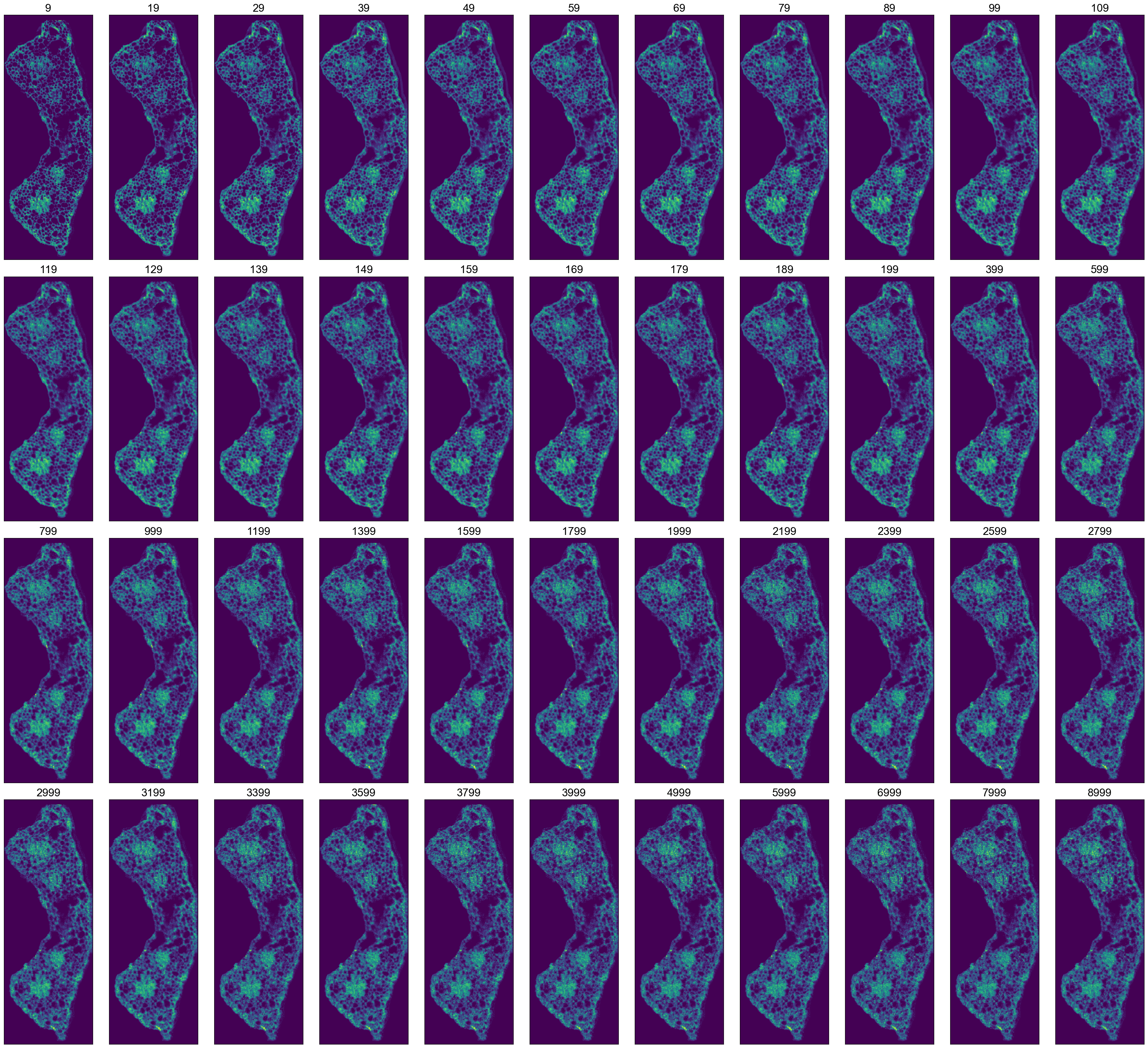


Figure S5. All checkpoint images that were used to calculate and plot Fig. 4b, 4c, 4d, and 4f.


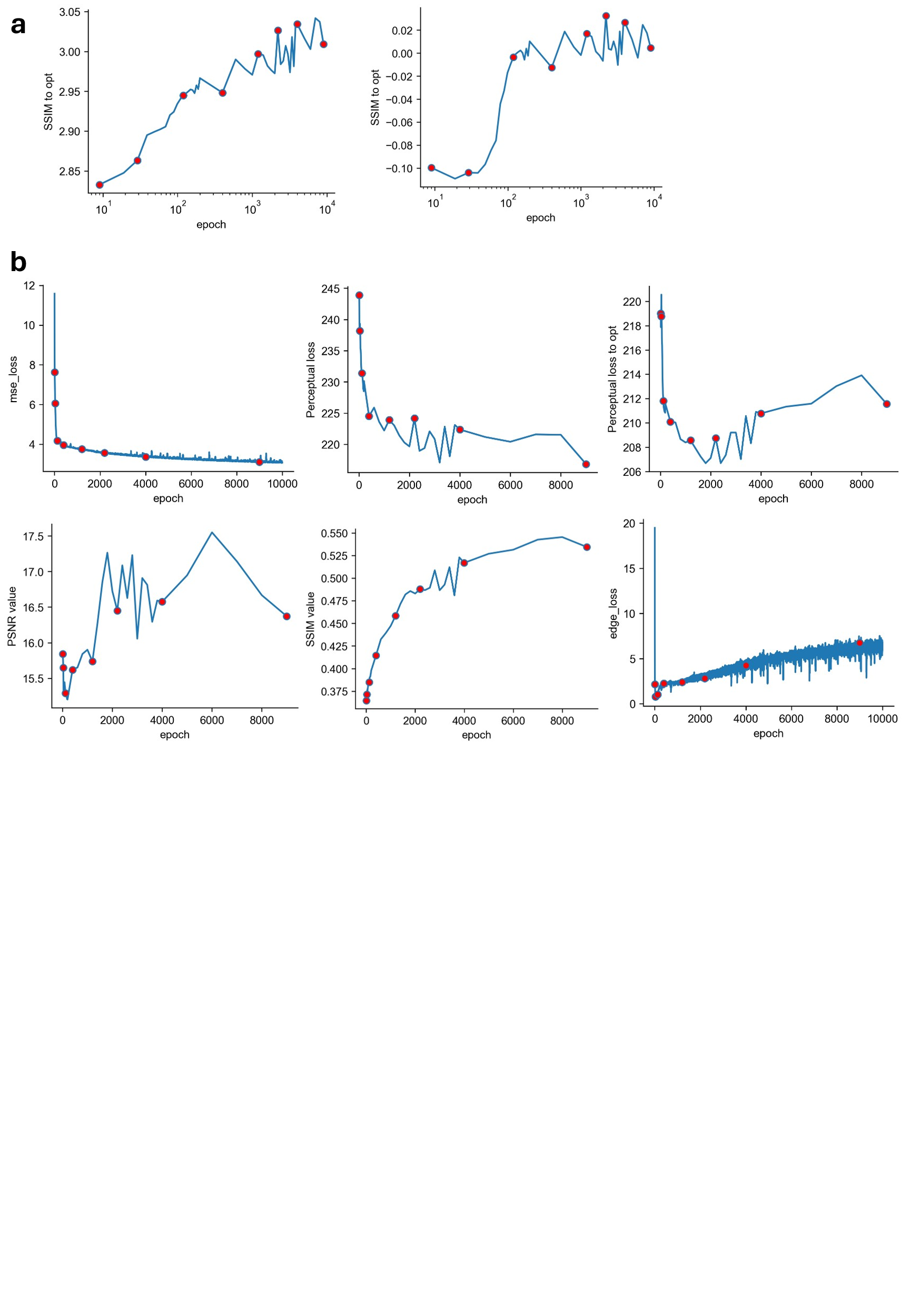


Figure S6. a) SSIM and PSNR calculated by the checkpoint images and the input microscopic image. b) Plots in Fig. 4 with normal linear axes.

Table S1. SSIM, PSNR, and perceptual loss (PLoss) values of fusion images from Fig. 2. Subscript indicates the referring image (Opt: microscopic image, MSI: ion image).

|  | SSIM_Opt_ | PSNR_Opt_ | SSIM_MSI_ | PSNR_MSI_ | PLoss_Opt_ | PLoss_MSI_ |
| --- | --- | --- | --- | --- | --- | --- |
| CNN | 0.03552 | 3.10402 | 0.48357 | 16.83111 | 174.918854 | 197.288712 |
| UNet | 0.008212 | 2.642607 | 0.640735 | 17.417324 | 5.615719 | 179.353668 |
| LCRN | -0.101688 | 2.983941 | 0.369539 | 15.152316 | 187.066116 | 212.679489 |

Table S2. SSIM, PSNR, and perceptual loss (PLoss) values of fusion images from Fig. 3. Subscript indicates the referring image (Opt: microscopic image, MSI: ion image).

|  | SSIM_Opt_ | PSNR_Opt_ | SSIM_MSI_ | PSNR_MSI_ | PLoss_Opt_ | PLoss_MSI_ |
| --- | --- | --- | --- | --- | --- | --- |
| CNN | 0.015607 | 3.886508 | 0.428764 | 16.114676 | 2276.254883 | 2121.510254 |
| UNet | 0.005743 | 3.891762 | 0.771586 | 22.690804 | 2511.114502 | 803.401489 |
| LCRN | 0.031136 | 3.865064 | 0.232889 | 13.968137 | 1884.003784 | 2163.560303 |
